# Differential gene expression in decidualized human endometrial stromal cells induced by different stimuli

**DOI:** 10.1038/s41598-024-58065-z

**Published:** 2024-04-02

**Authors:** Yumiko Doi-Tanaka, Isao Tamura, Amon Shiroshita, Taishi Fujimura, Yuichiro Shirafuta, Ryo Maekawa, Toshiaki Taketani, Shun Sato, Norihiro Sugino

**Affiliations:** Department of Obstetrics and Gynecology, Yamaguchi University Graduate School of Medicine, Minamikogushi 1-1-1, Ube, 755-8505 Japan

**Keywords:** Endocrinology, Endocrine system and metabolic diseases

## Abstract

Decidualization can be induced by culturing human endometrial stromal cells (ESCs) with several decidualization stimuli, such as cAMP, medroxyprogesterone acetate (MPA) or Estradiol (E_2_). However, it has been unclear how decidualized cells induced by different stimuli are different. We compared transcriptomes and cellular functions of decidualized ESCs induced by different stimuli (MPA, E_2_ + MPA, cAMP, and cAMP + MPA). We also investigated which decidualization stimulus induces a closer in vivo decidualization. Differentially expressed genes (DEGs) and altered cellular functions by each decidualization stimuli were identified by RNA-sequence and gene-ontology analysis. DEGs was about two times higher for stimuli that use cAMP (cAMP and cAMP + MPA) than for stimuli that did not use cAMP (MPA and E_2_ + MPA). cAMP-using stimuli altered the cellular functions including angiogenesis, inflammation, immune system, and embryo implantation whereas MPA-using stimuli (MPA, E_2_ + MPA, and cAMP + MPA) altered the cellular functions associated with insulin signaling. A public single-cell RNA-sequence data of the human endometrium was utilized to analyze in vivo decidualization. The altered cellular functions by in vivo decidualization were close to those observed by cAMP + MPA-induced decidualization. In conclusion, decidualized cells induced by different stimuli have different transcriptome and cellular functions. cAMP + MPA may induce a decidualization most closely to in vivo decidualization.

## Introduction

Implantation of the human embryo in the maternal endometrium is a key step in the establishment of pregnancy and requires a dialog between the embryo and the receptive endometrium^1^. Human endometrial stromal cells (ESCs) undergo cyclic changes during the menstrual cycle, including proliferation and differentiation that are controlled by estrogen and progesterone^2–5^. Decidualization is one of these changes in which ESCs are primed by estrogen in the proliferative phase, respond to progesterone, and become larger and rounder with significant changes in cellular functions. This process is critical for embryo implantation and the establishment of pregnancy. Impairment of decidualization leads to implantation failure, miscarriage, and unexplained infertility^6–8^.

Decidualization of ESCs has long been studied, mainly using in vitro models because it is impossible to study decidualization of ESCs directly in vivo for ethical reasons. Decidualization can be reproduced in vitro by culturing primary ESCs with medroxyprogesterone acetate (MPA) for 14 days^2,9^. As an alternative to MPA, cAMP has been widely used as a decidualization stimulus because cAMP is a second messenger of progesterone and can induce decidualization within a short period (4 days)^2,10^. In addition to these two protocols, other protocols have been established for in vitro decidualization. Estradiol (E_2_) may be added to MPA to mimic the secretion of progesterone and estrogen from the corpus luteum^2,11–16^. Another protocol is the combination of cAMP and MPA because it strongly induces decidualization^1^. Using these in vitro models, previous reports have revealed that decidualization is regulated by many factors, including intracellular signaling pathways, their related molecules, and transcription factors^9,17–22^. However, it should be noted that the decidualization stimuli were different among studies. Since insulin like growth factor binding protein 1 (IGFBP1) and prolactin (PRL) are specifically induced by the decidualization of ESCs^1,2,10,20,21,23,24^, in vitro decidualization has been assessed by the induction of IGFBP1 and PRL expressions. Although all decidualization protocols can induce their expressions, several reports pointed out that some gene expression patterns other than IGFBP1 and PRL were different among the protocols^19,25,26^. These facts led us to hypothesize that decidualized cells induced by different stimuli may not be the same, and if so, it is interesting to know how they differ.

Furthermore, it is important to consider whether in vitro decidualization can reproduce in vivo decidualization. Previous transcriptome analyses using human endometrium have identified differentially expressed genes in the late secretory phase endometrium compared with the proliferative phase endometrium^27,28^. However, these analyses do not identify the transcriptome changes specific to ESCs because the human endometrium is composed of a variety of cells. Therefore, it is unclear which gene expressions are altered in ESCs, and what kind of cellular functions are altered in ESCs by in vivo decidualization. A recent study with a single-cell RNA-sequence revealed the transcriptome of human endometrium at single-cell resolution across the menstrual cycle^29^. This makes it possible to identify the transcriptome changes in ESCs during in vivo decidualization. By comparing the transcriptome changes between in vivo and in vitro decidualization, we can identify the decidualization protocol that most closely reproduces in vivo decidualization.

In this study, we examined the transcriptomes and cellular functions of decidualized ESCs (dESCs) induced by different stimuli (MPA, E_2_ + MPA, cAMP, and cAMP + MPA). Furthermore, using a single-cell RNA-sequence data of the human endometrium, we investigated which in vitro decidualization stimulus most closely induces in vivo decidualization.

## Results

### Differential gene expressions by different decidualization stimuli

**Figure 1:**
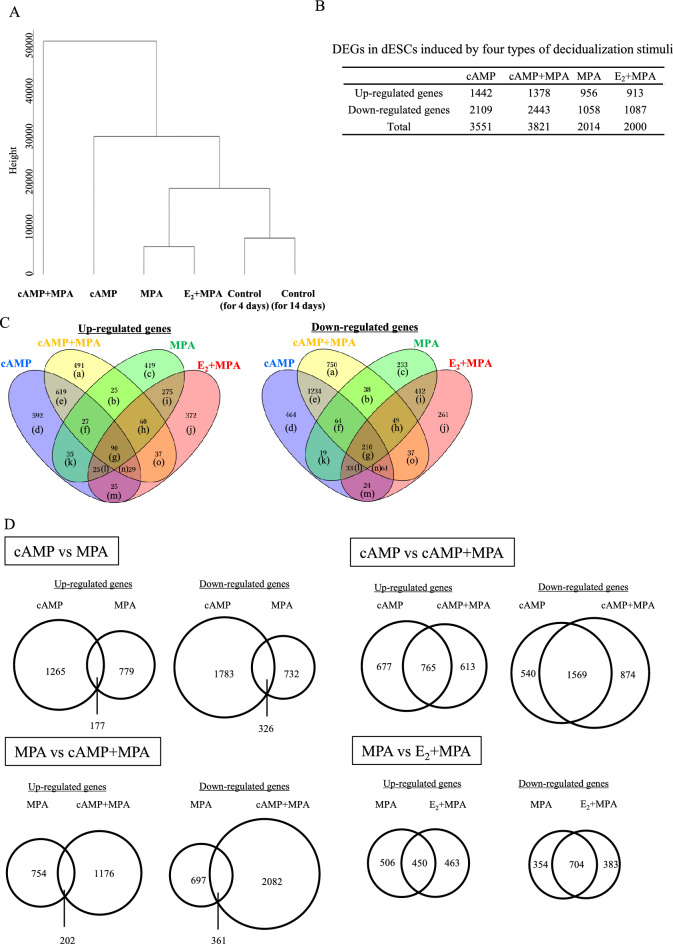
Differential gene expressions by different decidualization stimuli. (**A**) Hierarchical cluster analysis comparing the transcriptome of four types of dESCs induced by different stimuli (cAMP, cAMP + MPA, MPA, and E_2_ + MPA,) and the corresponding two control samples. Distances of gene expression pattern are indicated as height. (**B**) Numbers of DEGs in dESCs induced by four types of decidualization stimuli. (**C**) 4-way Venn diagrams comparing DEGs induced by four types of decidualization stimuli. Genes belonging to each compartment (a–o) are shown in Supplementary Table S2. (**D**) Venn diagrams indicating the number of specifically and commonly up- or down-regulated genes between decidualization stimuli.

We first confirmed that dESC samples used for RNA-sequence analysis showed the inductions of decidualization markers, IGFBP-1 and PRL (Supplementary Fig. S1). Then, we compared the transcriptomes of four types of dESCs induced by different stimuli (MPA, E_2_ + MPA, cAMP, and cAMP + MPA) and those of two corresponding control samples by a hierarchical clustering analysis (Fig. 1A). dESCs induced by any type of stimulus were classified separately from the control samples. Both cAMP and MPA can induce decidualization on their own, but were classified separately. As for the combined protocols, E_2_ + MPA was classified in the same cluster with MPA whereas cAMP + MPA were classified separately from cAMP. Regarding the distances from the controls, cAMP was located farther than the cluster of MPA and E_2+_MPA, and cAMP + MPA was further distant. These results suggested that the transcriptome profiles induced by MPA and cAMP were quite different and that cAMP induced a more significant change than MPA did. Furthermore, the addition of MPA to cAMP further changed the transcriptome whereas the addition of E_2_ to MPA did not induce a significant change.

To identify differentially expressed genes (DEGs) in dESCs induced by each stimulus, the transcriptomes of dESCs were compared with those of corresponding controls. The numbers of up-/down-regulated genes by each stimulus were as follows; cAMP:1442/2109, cAMP + MPA:1378/2443, MPA:956/1058, and E_2_ + MPA:913/1087 (Fig. 1B and Supplementary Table S2). The number of DEGs was about two times higher in cAMP-used stimuli (cAMP and cAMP + MPA) than in cAMP-non-used stimuli (MPA and E_2_ + MPA). We compared DEGs between four stimuli (Fig. 1C and Supplementary Table S2). Only 90 and 210 genes were identified as commonly up- or down-regulated genes, respectively (Fig. 1C, g). When DEGs were compared between cAMP and MPA, 177 genes were up-regulated by both cAMP and MPA, and 326 genes were down-regulated by both cAMP and MPA (Fig. 1D), showing that DEGs are quite different between cAMP and MPA stimuli. We also compared the DEGs between cAMP and cAMP + MPA (Fig. 1D). The addition of MPA to cAMP induced a number of different DEGs from those induced by cAMP. When compared between MPA and cAMP + MPA (Fig. 1D), the addition of cAMP to MPA induced a number of different DEGs from those induced by MPA. When compared between MPA and E2 + MPA (Fig. 1D), E2 induced a number of different DEGs from those induced by MPA.

### Differences in cellular functions altered in dESCs by different stimuli

**Figure 2:**
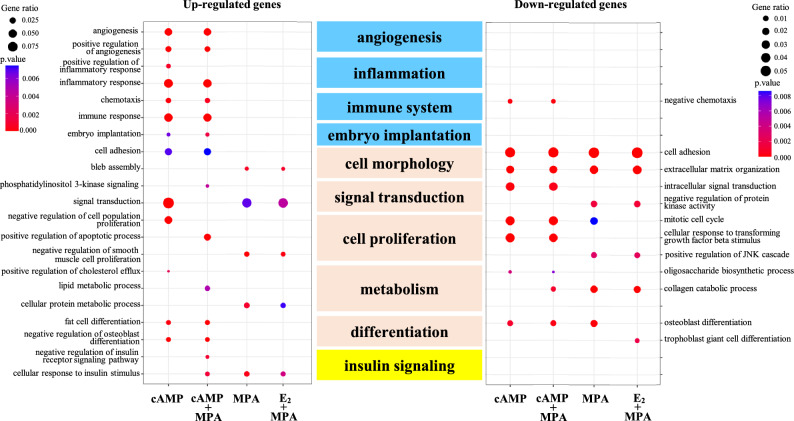
Differences in cellular functions altered in dESCs by different stimuli. The up- or down-regulated genes by each decidualization stimulus were subjected to GO-REVIGO analysis. The GO terms were classified into several groups according to their associated cellular functions (center column). Common cellular functions are indicated with orange rectangles. The cellular functions altered by cAMP-used stimuli and MPA-used stimuli are indicated with blue and yellow rectangles, respectively. The representative GO terms belonging to each cellular function are shown. The ratio of the number of identified genes to all genes in each term is designated as “Gene ratio” and is indicated with the size of the circle. P-values of each term are indicated with colors.

The different gene expression among decidualization stimuli led us to hypothesize that cellular functions altered in dESCs also depend on the stimulus. Therefore, to examine this, the up- and down-regulated genes were subjected to gene ontology (GO) analyses. A number of enriched terms were identified in the up- or down-regulated genes (Supplementary Tables S3 and S4). The enriched GO terms were then summarized by removing redundancy using reduce and visualize gene ontology (REVIGO), as reported previously^30,31^. The GO terms were classified into several groups according to their associated cellular functions (Supplementary Tables S3 and S4). The representative GO terms of these cellular functions are shown in Fig. 2. Gene ratio means the ratio of the number of identified genes to all genes in each term. They are indicated with the size of the circle. P-values of each term are indicated with colors. GO terms associated with ‘cell morphology’, ‘signal transduction’, ‘cell proliferation’, ‘metabolism’, and ‘differentiation’ (indicated with orange rectangles in Fig. 2) were detected in the up- and down-regulated genes by each of four stimuli. These cellular functions are designated as common functions among four stimuli. On the other hand, GO terms associated with ‘angiogenesis’, ‘inflammation’, ‘immune system’, and ‘embryo implantation’ (indicated with blue rectangles in Fig. 2) were mainly detected in the genes up-regulated by cAMP-using stimuli (cAMP and cAMP + MPA), but not by non-cAMP stimuli (MPA and E_2_ + MPA). GO terms associated with ‘insulin signaling’ (indicated with yellow rectangles in Fig. 2) were detected in the genes up-regulated by MPA-using stimuli (MPA, E_2_ + MPA, and cAMP + MPA), but not by non-MPA stimulus (cAMP). These cellular functions are designated as specific functions to cAMP-using stimuli or MPA-using stimuli.

**Figure 3:**
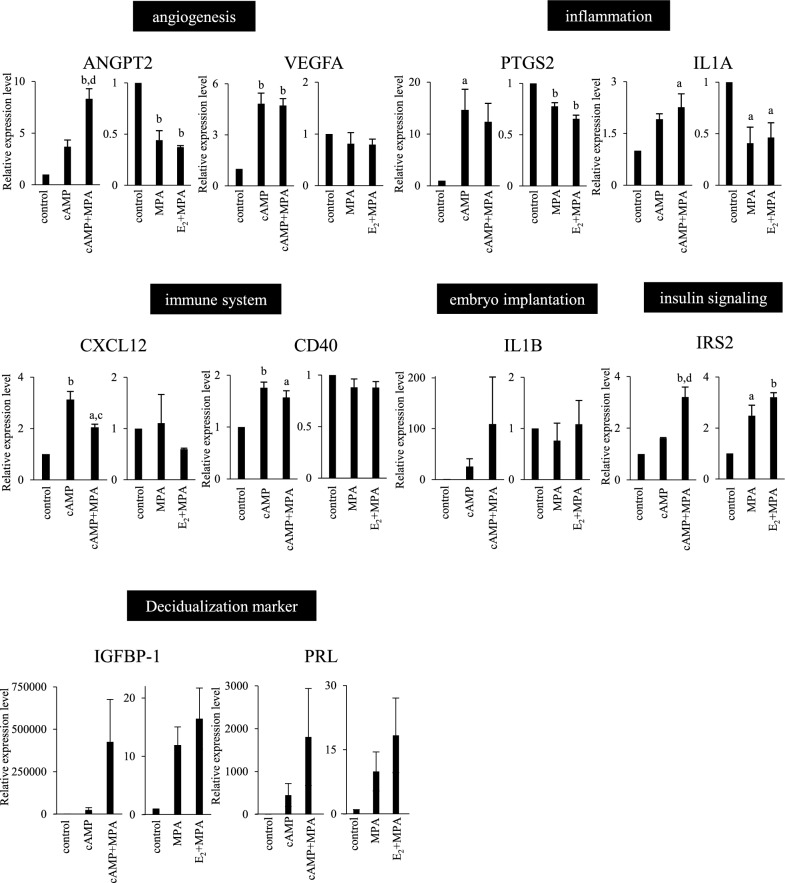
Validation of RNA-sequence data by real-time RT-PCR. ESCs were treated with the decidualization stimuli for 4 days (cAMP or cAMP + MPA) or 14 days (MPA or E_2_ + MPA). ESCs cultured without decidualization stimuli for 4 days or 14 days were used as the controls, respectively. We selected 8 genes that are the representative of the uncommon cellular functions. mRNA levels of these selected genes and decidualization markers (IGFBP-1 and PRL) were quantified by real-time RT-PCR. Values were normalized to those of MRPL19. The relative mRNA expression levels of each decidualized cells (cAMP, cAMP + MPA, MPA, E_2_ + MPA) were calculated as fold changes to the corresponding control cells. We repeated the three independent experiments on each three independent patient's cells. Then, the mean ± SE of fold change from three different individuals was used as a relative expression value in decidualized cells. Data are mean ± SE of three independent experiments. a, P < 0.05 vs. control; b, P < 0.01 vs. control; c, P < 0.05 vs. cAMP; d, P < 0.01 vs. cAMP.

The validation assay for RNA-sequence analysis was performed on genes that were associated with specific functions. For this purpose, we selected several genes belonging to gene ontology terms included in specific cellular functions (Supplementary Table S3, shown in bold). Their expression levels were examined by real-time RT-PCR (Fig. 3). We confirmed that dESCs samples used for RT-PCR showed the inductions of decidualization markers, IGFBP-1 and PRL. In agreement with the in-silico analysis, cAMP-using stimuli (cAMP and cAMP + MPA) up-regulated genes associated with angiogenesis (ANGPT2 and VEGFA), inflammation (PTGS2 and IL1A), immune system (CXCL12 and CD40) and embryo implantation (IL1B), whereas stimuli that did not contain cAMP (MPA and E_2_ + MPA) did not up-regulate them. On the other hand, MPA-using stimuli (MPA, E_2_ + MPA, and cAMP + MPA) up-regulated a gene associated with insulin signaling (IRS2), whereas a stimulus that did not contain MPA (cAMP) did not.

### Identification of DEGs by in vivo decidualization and their associated cellular functions

**Figure 4:**
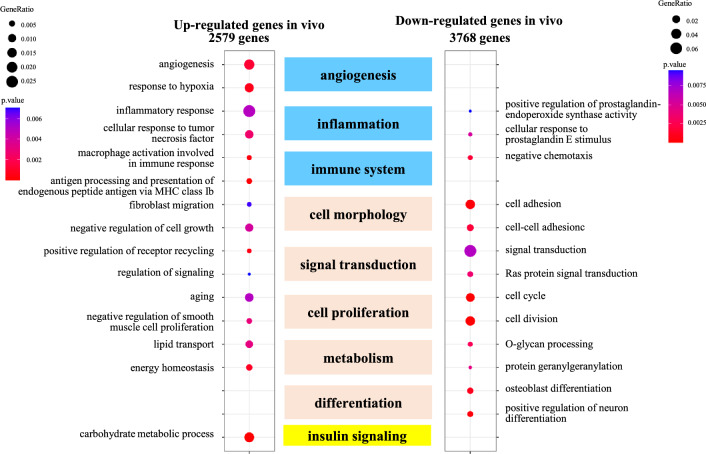
Cellular functions associated with DEGs by in vivo decidualization. By utilizing public single-cell RNA-sequence data of the human endometrium during the menstrual cycle, 2579 and 3768 genes were identified as the up- or down-regulated genes by in vivo decidualization, respectively. They were subjected to GO-REVIGO analysis. The GO terms were classified into several groups according to their associated cellular functions (center column). The representative GO terms belonging to each cellular function are shown. The ratio of the number of identified genes to all genes in each term is designated as “Gene ratio” and is indicated with the size of the circle. P-values of each term are indicated with colors.

We next investigated which stimulus most closely induces in vivo decidualization. To answer this question, we decided to identify the DEGs by in vivo decidualization using public single-cell RNA-sequence data of the human endometrium during the menstrual cycle^29^. The transcriptomes of 189 ESCs in the late proliferative phase (day 9 ~ day 11 of the menstrual cycle) were compared with those of 137 ESCs in the late secretory phase (day 24 ~ day 27 of the menstrual cycle) because the decidual reaction occurs in the late secretory phase endometrium^2^. The numbers of up-regulated and down-regulated genes by in vivo decidualization were 2579 and 3768 genes, respectively (Fig. 4 and Supplementary Table S5). The up- and down-regulated genes were subjected to GO-REVIGO analysis. GO terms were then classified into several cellular functions (Fig. 4 and Supplementary Table S6), which were used in the in vitro decidualization analysis (shown in Fig. 2). All of the common functions among four stimuli, which were observed by in vitro decidualization, were detected by in vivo decidualization. Among the specific functions induced by cAMP-using stimuli, ‘angiogenesis’, ‘inflammation’, and ‘immune system’ were also detected in in vivo decidualization. These cellular functions were mainly based on the up-regulated genes. On the other hand, ‘embryo implantation’ was not detected. A specific function induced by MPA-using stimuli, ‘insulin signaling’ was detected in in vivo decidualization, and it was based on the up-regulated genes. Thus, the cellular functions of dESCs induced by in vivo decidualization were close to those induced by cAMP + MPA in in vitro decidualization (Fig. 2).

### Comparison of DEGs between in vitro decidualization and in vivo decidualization

**Figure 5:**
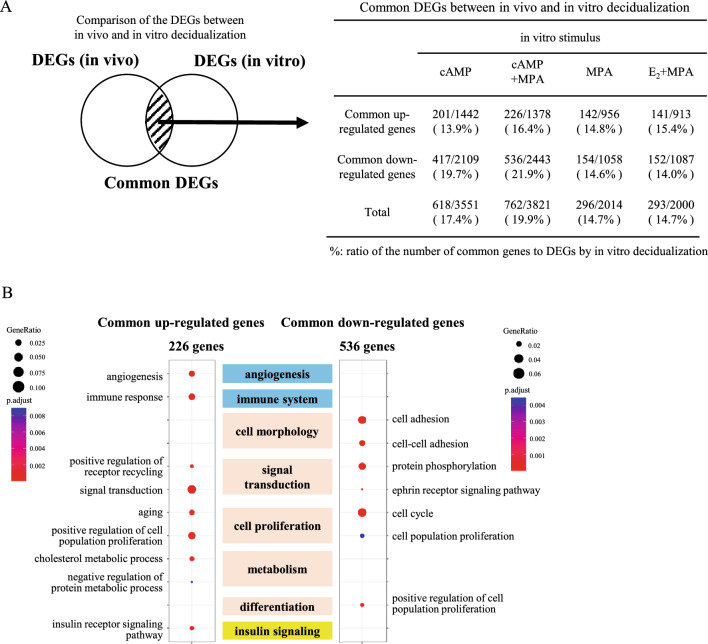
Comparison of DEGs and cellular functions between in vivo decidualization and in vitro decidualization. (**A**) DEGs by in vitro decidualization were compared with those by in vivo decidualization. The ratios of the number of common DEGs by each decidualization stimuli to DEGs by in vitro decidualization are shown as percentages. (**B**) Cellular functions associated with common DEGs by cAMP + MPA stimulus. 226 up-regulated genes and 536 down-regulated genes by cAMP + MPA stimulus were identified as common DEGs. They were subjected to GO-REVIGO analysis. The GO terms were classified into several groups according to their associated cellular functions (center column). The representative GO terms belonging to each cellular function are shown. The ratio of the number of identified genes to all genes in each term is designated as “Gene ratio” and is indicated with the size of the circle. P-values of each term are indicated with colors.

The DEGs shared between in vitro and in vivo decidualization are shown in Fig. 5A and Supplementary Table S7. Among four in vitro decidualization stimuli, cAMP + MPA stimulus had the higher number of common DEGs (762 genes), and the concordance ratio with in vivo decidualization was 19.9% (Fig. 5A).

We next examined cellular functions associated with the common 762 DEGs between cAMP + MPA-induced decidualization and in vivo decidualization (Fig. 5B, Supplementary Table S8). The up- or down-regulated genes in the common DEGs were subjected to GO-REVIGO analysis, and GO terms were then classified into several cellular functions. GO terms associated with ‘signal transduction’ and ‘cell proliferation’ were detected in the up- and down-regulated genes. GO terms associated with ‘angiogenesis’, ‘immune system’,‘metabolism’, and ‘insulin signaling’ were detected in the up-regulated genes while ‘cell morphology’ and ‘differentiation’ were detected in the down-regulated genes (Fig. 5B). The cellular function associated with ‘inflammation’ was not detected in the common DEGs. Twenty percent of the DEGs induced by cAMP + MPA were also induced in in vivo decidualization. The cellular functions associated with these DEGs included all cellular functions observed in in vivo decidualization except ‘inflammation’.

## Discussion

The present study showed that dESCs induced by different stimuli have quite different gene expression profiles and cellular functions. Several protocols have been used to induce decidualization so far^2^. Although cellular functions of dESCs have been reported to be different depending on the decidualization stimulus, the detailed profiles of dESCs induced by different stimuli remain unknown. This may have confused our understanding on in vitro decidualization. Therefore, our study provides important information about the detailed profiles including gene expressions and cellular functions of dESCs induced by different stimuli.

By analyzing the transcriptome of dESCs induced by different stimuli, we found that all stimulation protocols induced the cellular functions associated with cellular morphology, signal transduction, cell proliferation, metabolism, and differentiation, which are known to be important for decidualization^3–5^. Our results also showed that cAMP changed the expression of more genes than did MPA or E_2_ + MPA. Furthermore, in terms of cellular functions, well-known functions required for decidualization such as angiogenesis, immune system, inflammation, and embryo implantation^2,4^, were specifically induced when cAMP was used as a decidualization stimulus, but stimuli that did not use cAMP (MPA and E2 + MPA) did not. This is supported by previous reports showing that cAMP, but not MPA, up-regulates the expression of genes associated with these cellular functions^5,25,32–37^. cAMP is considered as a second messenger of progesterone because progesterone increases intracellular cAMP concentrations in ESCs^1,9^. Incubation of ESCs with cAMP rapidly increases intracellular cAMP levels and activates the signaling pathways downstream^1,14^. Therefore, cAMP takes 4 days to induce decidualization. On the other hand, decidualization induced by MPA (without cAMP) needs a longer period because the increase of intracellular cAMP takes more than 10 days^9,38^. cAMP has also been reported to induce significantly higher expression of decidualization markers than MPA does^1,9^, indicating that cAMP is a stronger decidualization stimulus than progesterone. Therefore, cAMP-using stimuli clearly altered more gene expressions and cellular functions than did MPA or E_2_ + MPA.

Interestingly, our result showed that the cellular functions associated with insulin signaling were induced only when MPA was used as a decidualization stimulus, while cAMP alone did not. One of the metabolisms that occur during decidualization is glucose metabolism, characterized by an increase in the storage of glycogen^39,40^. We and others have demonstrated that genes associated with the insulin signaling pathway, such as insulin receptor (INSR), insulin receptor substrate 1 (IRS1), and insulin receptor substrate 2 (IRS2), were up-regulated by E_2_ + MPA or cAMP + MPA, leading to an increase of glucose uptake^11,22,24,41–44^. Progesterone regulates gene transcription through its nuclear receptor, which acts as a ligand-dependent transcription factor^45^. Therefore, we speculate that insulin signaling is controlled by progesterone receptor (PR)-mediated regulation and is an MPA-specific cellular function that is not affected by cAMP. In fact, PR activated by MPA binds to the promoter region of IRS2 to increase its expression, which activates glucose uptake during decidualization^24,46^. Furthermore, the MPA-specific effects on gene regulation have been previously reported. Superoxide dismutase 1 (SOD1) was up-regulated in dESCs induced by E_2_ + MPA, but not by cAMP^47^. Heart and neural crest derivatives–expressed transcript 2 (HAND2), an important transcription factor for decidualization, is up-regulated in dESCs induced by E_2_ + MPA, but not by cAMP^48^. These facts support our current findings that certain gene expressions and cellular functions are altered by MPA, but not by cAMP.

The present study clearly showed that cAMP + MPA stimulus covered all functions altered by cAMP and MPA. It is reasonable to consider that this was due to a combination of the pathways activated by cAMP and MPA because MPA induced the expression of a number of different genes from those induced by cAMP (Fig. 1). In addition to the activation of each pathway, there are cross-talks between cAMP signaling and progesterone/PR signaling^1,2^. cAMP induces the expression of essential transcription factors for decidualization, including signal transducer and activator of transcription-5 (STAT5), CCAAT/enhancer-binding protein β (CEBPβ), and forkhead box protein O1A (FOXO1)^1,49,50^. These factors can increase the DNA binding activities of PR to enhance the expression of target genes during decidualization^46,51^. Furthermore, it has been reported that cAMP can enhance the transcriptional activity of PR by inhibiting the interaction with corepressors, such as nuclear corepressor (NCoR) and silencing mediator of retinoic acid and thyroid hormone receptor (SMRT), and by activating the interaction with coactivators, such as steroid receptor coactivator-1 (SRC-1) and CREB binding protein (CBP)^52–54^. On the contrary, progesterone/PR complex can modulate the transcriptional activities of critical transcription factors including STAT5, CEBPβ and FOXO1 for decidualization^49,55,56^. These interactions likely contribute to inducing multifunctional decidual phenotypes induced by cAMP + MPA.

Previous transcriptome analyses using the whole endometrium have attempted to identify DEGs by in vivo decidualization^27,28^. However, they have not been clarified due to the heterogeneity of the human endometrium. In this study, utilizing a public single-cell RNA-sequence data of human endometrium^29^, we have, for the first time, identified the changes in transcriptome and cellular functions specific to dESCs by in vivo decidualization. Our results indicate that 2579 genes were up-regulated and 3768 genes were down-regulated in ESCs during decidualization in human endometrium and that ESCs acquired several cellular functions during decidualization such as angiogenesis, inflammation, immune system, cell morphology, signal transduction, cell proliferation, metabolism, differentiation, and insulin signaling, which are known to be important for decidualization. Thus, the cellular functions of dESCs induced by in vivo decidualization were close to those observed by in vitro decidualization.

In terms of cellular functions, our results showed that cAMP + MPA induced a decidualization that most closely resembled in vivo decidualization. Thus, cAMP + MPA can reproduce changes in cellular function that are close to those in in vivo decidualization. The cellular function associated with embryo implantation was not observed in in vivo decidualization although it was observed in in vitro decidualization. This was not surprising since we analyzed the single-cell RNA-sequence data of ESCs derived from the late secretory phase endometrium, which is the period after implantation.

The present study showed that decidualized cells induced by different stimuli have quite different gene expression profiles and cellular functions. Among several protocols tried, cAMP + MPA most closely reproduces in vivo decidualization. However, the transcriptome changes induced by in vitro decidualization may be different from those induced by in vivo decidualization, which should be kept in our mind when we interpret the results obtained from in vitro decidualization. The present results should be very useful for designing future studies of decidualization, as well as for better understanding the previous studies.

## Materials and methods

### Reagents

Dulbecco’s modified Eagle medium (DMEM), L-glutamine, 1 × trypsin–EDTA, streptomycin, and penicillin were purchased from Invitrogen (Carlsbad, CA, USA). Fetal bovine serum (FBS) was obtained from Biological Industries Ltd (Beit Haemek, Israel). Collagenases, dibutyryl cyclic adenosine monophosphate (cAMP), Estradiol (E_2_) and medroxyprogesterone acetate (MPA) were obtained from Sigma Chemical Co Ltd.

### ESC isolation

Human endometrial tissues were obtained at hysterectomy from patients with a normal menstrual cycle, aged 40–45 years, who underwent surgery for myoma uteri. The patients were not on hormonal therapy at the time of surgery. Informed consent was obtained from all participating patients, and ethical approval was obtained from the Institutional Review Board of Yamaguchi University Hospital (H26-102–7). All experiments were performed in accordance with the Tenets of the Declaration of Helsinki. Endometrial samples utilized for ESC isolation were histologically diagnosed as being in the late proliferative phase according to the published criteria^57^. Tissue samples were washed with Phenol Red-free DMEM containing 4 mM glutamine, 50 mg/ml streptomycin and 50 IU/ml penicillin, and minced into pieces of < 1 mm^3^. ESCs were isolated as reported previously^58^. Cells were seeded at 10^5^ cells/cm^2^ in 75 cm^2^ tissue culture flasks and incubated in Phenol Red-free DMEM containing glutamine, antibiotics, and 10% dextran-coated charcoal-stripped FBS at 37 °C, 95% air and 5% CO_2_. At confluence, cells were treated with 1 × trypsin–EDTA and subcultured to use each experiment.

### Induction of in vitro decidualization

To induce decidualization, ESCs from one patient were subcultured into 6 groups and were incubated with treatment medium (Phenol Red-free DMEM supplemented with glutamine, antibiotics and 2% dextran-coated charcoal-stripped FBS) with or without decidualization stimuli. Decidualization can be induced by culturing ESCs with cAMP (0.5 mM) for 4 days or MPA (10^−6^ M) for 14 days. These are both considered the basic protocols for decidualization induction because MPA and cAMP are the main decidualization inducers^1^. In addition to these basic protocols, E_2_ (10^−8^ M) may be added to MPA for 14 days (E_2_ + MPA), or MPA may be added to cAMP for 4 days (cAMP + MPA)^2^. These combined stimuli have been widely used as well. Therefore, we prepared dESCs induced by four types of stimulation protocols (cAMP, cAMP + MPA, MPA, and E_2_ + MPA). Because the cultured period was different between the 4-days protocol (cAMP and cAMP + MPA) and the 14-days protocol (MPA, and E_2_ + MPA), we prepared two control ESCs (non-dESCs) that were cultured without stimulations for 4 days or 14 days, respectively. The concentration of decidualization stimuli and the period of incubation used in this study were based on our previous report^9,11,50^. The medium was changed every other day.

### Whole-transcriptome analysis with RNA sequence

ESCs from one patient, who underwent surgery for myoma uteri, were subcultured into 6 groups (control sample for the 4-days protocol, cAMP, cAMP + MPA, control sample for the 14-days protocol, MPA, and E_2_ + MPA). Total RNA was isolated from the cultured cells with an RNeasy® Mini Kit with DNAse treatment (QIAGEN, Inc., Valencia, CA). RNA-sequence was performed as reported previously^59–61^. mRNA was purified with oligo dT beads (NEBNext Poly(A) mRNA magnet Isolation Module, New England Biolabs). Complementary DNA libraries for Illumina sequencing were generated with NEBNext Ultra II RNA library Prep kit (NEB) and NEBNextplex Oligos for Illumina. The confirmed libraries were sequenced with Illumina Next-seq DNA sequencer with a 75-bp pair-end cycle sequencing kit (Illumina). To produce the raw bcl, or base call files, quality assessment and image analyses were performed using Next-seq packaging software (Illumina) Real Time Analysis, and bcl2fastaq Conversion Software v2.19 (Illumina) was used for demultiplexing of the samples and adaptor removable. Reads with more than two ambiguous nucleotides and reads with quality scores less than 13 as calculated by the Phred program were removed using CLC Genomics Workbench software (version 12.0.3, QIAGEN). The DNA fragments were read with 75-bp pair-end cycle sequencing. After mapping the paired reads, if they were more than 1000 bases apart or less than 20 bases close, they were discarded. Trimmed reads were mapped to the human reference genome GRCh37 (version 19) with CLC Genomics Workbench software (version 12.0.3) in default settings. Briefly, the reads were aligned to reference using the setting conditions with mismatch cost of 2, insertion cost of 3, and deletion cost of 3. In addition, the reads were mapped when at least 80% of the alignment matched the reference sequence (length fraction of 0.8), and the matched alignment was at least 80% identical to the reference sequences (similarity fraction of 0.8). Gene expression values were calculated as “transcripts per million” (TPM) as reported previously^62^. 1 was added to the TPM value before the following calculation. When the TPM value increased or decreased more than 2.0-fold or 0.5-fold compared to the corresponding control samples, these genes were defined as up- or downregulated genes by in vitro decidualization.

### In-silico analysis of single-cell RNA-sequence data

To investigate the transcriptome changes in ESCs during in vivo decidualization, we utilized public single-cell RNA-sequence data of the human endometrium during the menstrual cycle (GEO accession: GSE111976)^29^. In their study, dimensional reduction via t-distributed stochastic neighbor embedding (t-SNE) was performed to segregate the cells into distinct groups. According to the canonical markers and highly differentially expressed genes, one of the cell populations was identified as ESCs. Therefore, cells determined as ESCs were used for our analysis. We extracted and analyzed the transcriptome of ESCs by Seurat (version 4.0.3). Then, the transcriptomes of 189 ESCs from 4 patients in the late proliferative phase (day 9 ~ day 11 of the menstrual cycle) were compared with those of 137 ESCs from 5 patients in the late secretory phase (day 24 ~ day 27 of the menstrual cycle) that express FOXO1, one of the decidualization markers^1,63^. To determine the expression level of each gene, the mean TPM of all cells in the late proliferative and secretory phase were calculated, respectively. These values were used as the gene expression levels for each phase. 1 was added to mean TPM before the following calculation. When gene expression levels in the late secretory phase increased or decreased more than 2.0-fold or 0.5-fold compared to those in the late proliferative phase, these genes were defined as up- or down-regulated genes by in vivo decidualization, respectively.

### Real-time RT-PCR

ESCs from one patient were subcultured into 6 groups ESCs (control for day 4, cAMP, cAMP + MPA, control for day14, MPA, E + MPA). Total RNA was isolated with an RNeasy Mini Kit with DNase treatment (QIAGEN). RT reaction and real-time RT-PCR were performed as reported previously^58^, using CFX384 Real-Time System (Bio-Rad, Hercules, CA) with Luna Universal qPCR Master Mix (Bio-Rad). The primer sequences are shown in Supplementary Table S1. Mitochondrial ribosomal protein L19 (MRPL19) were used as internal controls as reported previously^64,65^. The relative mRNA expression levels of each decidualized cells (cAMP, cAMP + MPA, MPA, E + MPA) were calculated as fold changes to the corresponding control cells. This is a result from an individual ESCs. We repeated the three independent experiments on each three independent patient's cells. Then, the mean ± SE of fold change from three different individuals was used as a relative expression value in decidualized cells.

### Bioinformatics

A hierarchical clustering analysis was performed using the R package “Cluster” (version 2.1.4)^66^. DAVID Bioinformatics Resources (version 6.8) (https://david.ncifcrf.gov/) was used to determine whether the functional annotation of the differentially expressed genes was enriched for specific Gene Ontology (GO) terms^67^. P values less than 0.01 were considered to indicate significant enrichment. Then, the enriched GO terms were summarized by removing redundancy and plotted using reduce and visualize gene ontology (REVIGO) with allowed similarity as “small (0.5)” as reported previously^30,31,68^.

### Statistical analysis

To analyze differences between groups, statistical significance was analyzed by a Tukey–Kramer test. All statistical analyses were performed using R (version 4.0.2, R Foundation for Statistical Computing, Vienna, Austria). Differences were considered significant at *P* < 0.05.

### Supplementary Information

Supplementary Figure 1.

Supplementary Table 1.

Supplementary Table 2.

Supplementary Table 3.

Supplementary Table 4.

Supplementary Table 5.

Supplementary Table 6.

Supplementary Table 7.

Supplementary Table 8.

## Data Availability

Original data generated and analyzed during this study are included in this published article or in the data repositories listed in References. RNA-seq data are deposited in Gene Expression Omnibus (GEO) (GEO accession no.: GSE235151).

## References

[1] Gellersen B, Brosens J (2003). Cyclic AMP and progesterone receptor cross-talk in human endometrium: A decidualizing affair. J. Endocrinol..

[2] Gellersen B, Brosens J (2014). Cyclic decidualization of the human endometrium in reproductive health and failure. Endocr. Rev..

[3] Zhu H, Hou CC, Luo LF, Hu YJ, Yang WX (2014). Endometrial stromal cells and decidualized stromal cells: Origins, transformation and functions. Gene..

[4] Gellersen B, Brosens IA, Brosens JJ (2007). Decidualization of the human endometrium: mechanisms, functions, and clinical perspectives. Semin. Reprod. Med..

[5] Murata H, Tanaka S, Okada H (2022). The regulators of human endometrial stromal cell decidualization. Biomolecules..

[6] Salker M (2010). Natural selection of human embryos: impaired decidualization of endometrium disables embryo-maternal interactions and causes recurrent pregnancy loss. PLoS One..

[7] Laird SM, Tuckerman EM, Li TC (2006). Cytokine expression in the endometrium of women with implantation failure and recurrent miscarriage. Reprod. Biomed. Online..

[8] Ticconi C, Di Simone N, Campagnolo L, Fazleabas A (2021). Clinical consequences of defective decidualization. Tissue Cell..

[9] Matsuoka A (2010). Progesterone increases manganese superoxide dismutase expression via a cAMP-dependent signaling mediated by noncanonical Wnt5a pathway in human endometrial stromal cells. J. Clin. Endocrinol. Metab..

[10] Tamura I (2012). Induction of IGFBP-1 expression by cAMP is associated with histone acetylation status of the promoter region in human endometrial stromal cells. Endocrinology..

[11] Tamura I (2014). Genome-wide analysis of histone modifications in human endometrial stromal cells. Mol. Endocrinol..

[12] Murata, H. *et al.* Transcriptional regulation of LGALS9 by HAND2 and FOXO1 in human endometrial stromal cells in women with regular cycles. *Mol. Hum. Reprod.* **27**, gaab063 (2021). 10.1093/molehr/gaab063 34581822

[13] Maekawa R (2019). Genome-wide DNA methylation analysis revealed stable DNA methylation status during decidualization in human endometrial stromal cells. BMC Genomics..

[14] Tamura I (2020). Wilms tumor 1 regulates lipid accumulation in human endometrial stromal cells during decidualization. J. Biol. Chem..

[15] Tamura, I. *et al.* Genome-wide analysis of histone modifications that underlie the dynamic changes in gene expression during decidualization in human endometrial stromal cells. *Mol. Hum. Reprod.* **29**, gaad019 (2023). 10.1093/molehr/gaad019 37310913

[16] Kida N (2021). Exposure to cigarette smoke affects endometrial maturation including angiogenesis and decidualization. Reprod. Med. Biol..

[17] Wang W, Taylor RN, Bagchi IC, Bagchi MK (2012). Regulation of human endometrial stromal proliferation and differentiation by C/EBPbeta involves cyclin E-cdk2 and STAT3. Mol Endocrinol..

[18] Aghajanova L (2010). The protein kinase A pathway-regulated transcriptome of endometrial stromal fibroblasts reveals compromised differentiation and persistent proliferative potential in endometriosis. Endocrinology..

[19] Popovici RM, Kao LC, Giudice LC (2000). Discovery of new inducible genes in in vitro decidualized human endometrial stromal cells using microarray technology. Endocrinology..

[20] Tamura I (2014). Importance of C/EBPbeta binding and histone acetylation status in the promoter regions for induction of IGFBP-1, PRL, and Mn-SOD by cAMP in human endometrial stromal cells. Endocrinology..

[21] Tamura I (2017). Novel function of a transcription factor WT1 in regulating decidualization in human endometrial stromal cells and its molecular mechanism. Endocrinology..

[22] Jozaki K (2019). Glucose regulates the histone acetylation of gene promoters in decidualizing stromal cells. Reproduction..

[23] Tamura I (2018). The distal upstream region of insulin-like growth factor-binding protein-1 enhances its expression in endometrial stromal cells during decidualization. J. Biol. Chem..

[24] Neff, A. M., Yu, J., Taylor, R. N., Bagchi, I. C. & Bagchi, M. K. Insulin signaling via progesterone-regulated insulin receptor substrate 2 is critical for human uterine decidualization. *Endocrinology.* **161**, bpz021 (2020). 10.1210/endocr/bqz021 PMC6986554 31748790

[25] Murata H (2019). Progestin-induced heart and neural crest derivatives-expressed transcript 2 inhibits angiopoietin 2 via fibroblast growth factor 9 in human endometrial stromal cells. Reprod. Biol..

[26] Cho, H. *et al.* Progestin-induced heart and neural crest derivatives expressed transcript 2 is associated with fibulin-1 expression in human endometrial stromal cells. *Fertil. Steril.* **99**, 248–255 e242 (2013). 10.1016/j.fertnstert.2012.08.056 23036802

[27] Talbi S (2006). Molecular phenotyping of human endometrium distinguishes menstrual cycle phases and underlying biological processes in normo-ovulatory women. Endocrinology..

[28] Ponnampalam AP, Weston GC, Trajstman AC, Susil B, Rogers PA (2004). Molecular classification of human endometrial cycle stages by transcriptional profiling. Mol. Hum. Reprod..

[29] Wang W (2020). Single-cell transcriptomic atlas of the human endometrium during the menstrual cycle. Nat. Med..

[30] Shirafuta, Y. *et al.* Integrated analysis of transcriptome and histone modifications in granulosa cells during ovulation in female mice. *Endocrinology.* **162**, bqab128 (2021). 10.1210/endocr/bqab128 34171084

[31] Maekawa R (2022). Different DNA methylome, transcriptome and histological features in uterine fibroids with and without MED12 mutations. Sci. Rep..

[32] Okada H (2014). Regulation of decidualization and angiogenesis in the human endometrium: Mini review. J. Obstet. Gynaecol. Res..

[33] Tsuzuki T (2013). Divergent regulation of angiopoietin-1, angiopoietin-2, and vascular endothelial growth factor by hypoxia and female sex steroids in human endometrial stromal cells. Eur. J. Obstet. Gynecol. Reprod. Biol..

[34] Matsui N, Kawano Y, Nakamura S, Miyakawa I (2004). Changes in vascular endothelial growth factor production associated with decidualization by human endometrial stromal cells in vitro. Acta Obstet. Gynecol. Scand..

[35] Yu J (2020). Human endometrial stromal cell differentiation is stimulated by PPARbeta/delta activation: New targets for infertility?. J. Clin. Endocrinol. Metab..

[36] Tamura M (2002). Interleukin-1beta elevates cyclooxygenase-2 protein level and enzyme activity via increasing its mRNA stability in human endometrial stromal cells: An effect mediated by extracellularly regulated kinases 1 and 2. J. Clin. Endocrinol. Metab..

[37] Zhao D, Lebovic DI, Taylor RN (2002). Long-term progestin treatment inhibits RANTES (regulated on activation, normal T cell expressed and secreted) gene expression in human endometrial stromal cells. J. Clin. Endocrinol. Metab..

[38] Brar AK, Frank GR, Kessler CA, Cedars MI, Handwerger S (1997). Progesterone-dependent decidualization of the human endometrium is mediated by cAMP. Endocrine..

[39] Dean M (2019). Glycogen in the uterus and fallopian tubes is an important source of glucose during early pregnancydagger. Biol. Reprod..

[40] Tamura I (2023). Glucose and lipid metabolisms in human endometrial stromal cells during decidualization. Endocr J..

[41] Tamura, I. *et al.* The essential glucose transporter GLUT1 is epigenetically upregulated by C/EBPbeta and WT1 during decidualization of the endometrium. *J. Biol. Chem.* 101150 (2021). 10.1016/j.jbc.2021.101150 PMC8458984 34478711

[42] Frolova A (2009). Facilitative glucose transporter type 1 is differentially regulated by progesterone and estrogen in murine and human endometrial stromal cells. Endocrinology..

[43] Frolova AI, Moley KH (2011). Quantitative analysis of glucose transporter mRNAs in endometrial stromal cells reveals critical role of GLUT1 in uterine receptivity. Endocrinology..

[44] Thirone AC, Huang C, Klip A (2006). Tissue-specific roles of IRS proteins in insulin signaling and glucose transport. Trends Endocrinol. Metab..

[45] Ng SW (2020). Endometrial decidualization: The primary driver of pregnancy health. Int. J. Mol. Sci..

[46] Kaya HS (2015). Roles of progesterone receptor A and B isoforms during human endometrial decidualization. Mol. Endocrinol..

[47] Sugino N, Karube-Harada A, Sakata A, Takiguchi S, Kato H (2002). Different mechanisms for the induction of copper-zinc superoxide dismutase and manganese superoxide dismutase by progesterone in human endometrial stromal cells. Hum. Reprod..

[48] Shindoh H, Okada H, Tsuzuki T, Nishigaki A, Kanzaki H (2014). Requirement of heart and neural crest derivatives-expressed transcript 2 during decidualization of human endometrial stromal cells in vitro. Fertil. Steril..

[49] Takano M (2007). Transcriptional cross talk between the forkhead transcription factor forkhead box O1A and the progesterone receptor coordinates cell cycle regulation and differentiation in human endometrial stromal cells. Mol. Endocrinol..

[50] Tamura I (2021). Transcription factor C/EBPbeta induces genome-wide H3K27ac and upregulates gene expression during decidualization of human endometrial stromal cells. Mol. Cell Endocrinol..

[51] Vasquez YM (2015). FOXO1 is required for binding of PR on IRF4, novel transcriptional regulator of endometrial stromal decidualization. Mol. Endocrinol..

[52] Rowan BG, Garrison N, Weigel NL, O'Malley BW (2000). 8-Bromo-cyclic AMP induces phosphorylation of two sites in SRC-1 that facilitate ligand-independent activation of the chicken progesterone receptor and are critical for functional cooperation between SRC-1 and CREB binding protein. Mol. Cell Biol..

[53] Rowan, B. G., Weigel, N. L. & O'Malley, B. W. Phosphorylation of steroid receptor coactivator-1. Identification of the phosphorylation sites and phosphorylation through the mitogen-activated protein kinase pathway. *J. Biol. Chem.* **275**, 4475–4483 (2000). 10.1074/jbc.275.6.4475 10660621

[54] Wagner BL, Norris JD, Knotts TA, Weigel NL, McDonnell DP (1998). The nuclear corepressors NCoR and SMRT are key regulators of both ligand- and 8-bromo-cyclic AMP-dependent transcriptional activity of the human progesterone receptor. Mol. Cell Biol..

[55] Lee JH (2013). Signal transducer and activator of transcription-3 (Stat3) plays a critical role in implantation via progesterone receptor in uterus. FASEB J..

[56] Christian M, Pohnke Y, Kempf R, Gellersen B, Brosens JJ (2002). Functional association of PR and CCAAT/enhancer-binding protein beta isoforms: promoter-dependent cooperation between PR-B and liver-enriched inhibitory protein, or liver-enriched activatory protein and PR-A in human endometrial stromal cells. Mol. Endocrinol..

[57] Noyes RW, Hertig AT, Rock J (1975). Dating the endometrial biopsy. Am. J. Obstet. Gynecol..

[58] Tamura I (2011). Differential effects of progesterone on COX-2 and Mn-SOD expressions are associated with histone acetylation status of the promoter region in human endometrial stromal cells. J. Clin. Endocrinol. Metab..

[59] Takagi H (2022). Transcriptional coactivator PGC-1alpha contributes to decidualization by forming a histone-modifying complex with C/EBPbeta and p300. J. Biol. Chem..

[60] Watanabe K (2018). A novel somatic mutation of SIN3A detected in breast cancer by whole-exome sequencing enhances cell proliferation through ERalpha expression. Sci. Rep..

[61] Tamura I (2022). Effects of melatonin on the transcriptome of human granulosa cells, fertilization and blastocyst formation. Int. J. Mol. Sci..

[62] Li B, Ruotti V, Stewart RM, Thomson JA, Dewey CN (2010). RNA-Seq gene expression estimation with read mapping uncertainty. Bioinformatics..

[63] Adiguzel D, Celik-Ozenci C (2021). FoxO1 is a cell-specific core transcription factor for endometrial remodeling and homeostasis during menstrual cycle and early pregnancy. Hum. Reprod. Update..

[64] Ayakannu T (2015). Validation of endogenous control reference genes for normalizing gene expression studies in endometrial carcinoma. Mol. Hum. Reprod..

[65] Ham S, Harrison C, Southwick G, Temple-Smith P (2016). Selection of internal control genes for analysis of gene expression in normal and diseased human dermal fibroblasts using quantitative real-time PCR. Exp. Dermatol..

[66] Maechler, M., Rousseeuw, P., Struyf, A., Hubert, M. & Hornik, K. cluster: Cluster Analysis Basics and Extensions. R package version 2.1.4—For new features, see the 'Changelog' file (in the package source), https://CRAN.R-project.org/package=cluster. (2022).

[67] Huang DW (2007). DAVID Bioinformatics Resources: Expanded annotation database and novel algorithms to better extract biology from large gene lists. Nucleic Acids Res..

[68] Supek F, Bosnjak M, Skunca N, Smuc T (2011). REVIGO summarizes and visualizes long lists of gene ontology terms. PLoS One..

