# Supplementary material for: Differential gene expression in decidualized human endometrial stromal cells induced by different stimuli
Source: Sci Rep. 2024 Apr 2;14:7726. doi: 10.1038/s41598-024-58065-z (PMC10987566; doi:10.1038/s41598-024-58065-z)
Supplement: Supplementary file 1 — Supplementary Figure 1. [file 41598_2024_58065_MOESM1_ESM.docx]

Supporting Information for

**Differential gene expression in decidualized human endometrial stromal cells induced by different stimuli**

Yumiko Doi-Tanaka, Isao Tamura*, Amon Shiroshita, Taishi Fujimura, Yuichiro Shirafuta, Ryo Maekawa, Toshiaki Taketani, Shun Sato, Norihiro Sugino

Department of Obstetrics and Gynecology, Yamaguchi University Graduate School of Medicine, Minamikogushi 1-1-1, Ube, 755-8505 Japan

**Contents of this file**


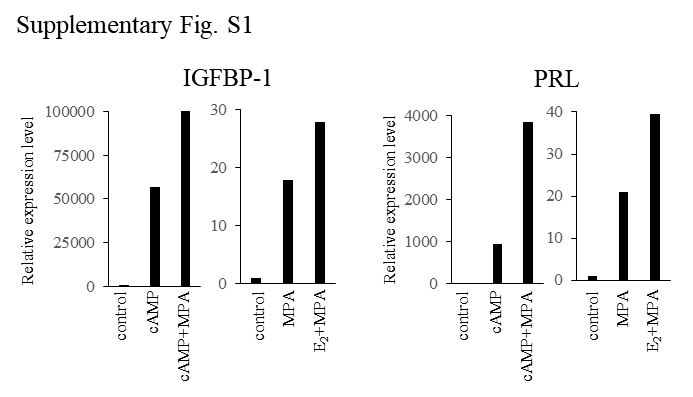
・Supplementary Fig. S1 and its legend

**Supplementary Figure S1. Induction of decidualization markers in decidualized ESCs used for RNA-sequence.**

mRNA levels of decidualization markers (IGFBP-1 and PRL) in the samples used for RNA-sequence analysis were quantified by real-time RT-PCR. Values were normalized to those of MRPL19. The relative mRNA expression levels of each decidualized cells (cAMP, cAMP+MPA, MPA, E_2_+MPA) were calculated as fold changes to the corresponding control cells.
